# *In Vitro* Effects of Wistar Rat Prenatal and Postnatal Cerebrospinal Fluid on Neural Differentiation and Proliferation of Mesenchymal Stromal Cells Derived from Bone Marrow

**DOI:** 10.22074/cellj.2018.4130

**Published:** 2017-11-04

**Authors:** Rozmehr Shokohi, Mohammad Nabiuni, Saeed Irian, Jaleel A. Miyan

**Affiliations:** 1 Department of Cell and Molecular Biology, Faculty of Biological Sciences, Kharazmi University, Tehran, Iran; 2 Faculty of Life Sciences, The University of Manchester, Manchester, UK

**Keywords:** Cell Proliferation, Cerebrospinal Fluid, Mesenchymal Stromal Cells, Neural Differentiation

## Abstract

**Objective:**

Cerebrospinal fluid (CSF) plays an important role in cortical development during the fetal stages. Embryonic CSF (E-CSF) consists of numerous neurotrophic and growth factors that regulate neurogenesis, differentiation, and proliferation. Mesenchymal stem cells (MSCs) are multi-potential stem cells that can differentiate into mesenchymal and non-mesenchymal cells, including neural cells. This study evaluates the prenatal and postnatal effects of CSF on proliferation and neural differentiation of bone marrow MSCs (BM-MSCs) at gestational ages E19, E20, and the first day after birth (P1).

**Materials and Methods:**

In this experimental study, we confirmed the mesenchymal nature of BM-MSCs according to their adherence properties and surface markers (CD44, CD73 and CD45). The multi-potential characteristics of BM- MSCs were verified by assessments of the osteogenic and adipogenic potentials of these cells. Under appropriate in vitro conditions, the BM-MSCs cultures were incubated with and without additional pre- and postnatal CSF. The MTT assay was used to quantify cellular proliferation and viability. Immunocytochemistry was used to study the expression of MAP-2 and β-III tubulin in the BM-MSCs. We used ImageJ software to measure the length of the neurites in the cultured cells.

**Results:**

BM-MSCs differentiated into neuronal cell types when exposed to basic fibroblast growth factor (b-FGF). Viability and proliferation of the BM-MSCs conditioned with E19, E20, and P1 CSF increased compared to the control group. We observed significantly elevated neural differentiation of the BM-MSCS cultured in the CSF-supplemented medium from E19 compared to cultures conditioned with E20 and P1 CSF group.

**Conclusion:**

The results have confirmed that E19, E20, and P1 CSF could induce proliferation and differentiation of BM-MSCs though they are age dependent factors. The presented data support a significant, conductive role of CSF components in neuronal survival, proliferation, and differentiation.

## Introduction

The vertebrate central nervous system (CNS) develops from the neural tube filled with embryonic cerebrospinal fluid (E-CSF) and neuroepithelial cells adjacent to the E-CSF (1). CSF is mainly produced by the choroid plexus cells in the brain ventricles. During development and early postnatal stages, the CSF contains a high concentration of proteins in contrast to its low protein content in normal adults (2, 3). Evidence suggests that the E-CSF contains diffusible factors such as transforming growth factor-β (TGF-β), nerve growth factor (NGF), brain derived neurotrophic factor (BDNF), neurotrophin-3 (NT-3), insulin-like growth factor (IGF), hepatocyte growth factor (HGF), and basic fibroblast growth factor (b-FGF). All of these factors are important for neurogenesis regulation (4, 5). b-FGF in the E-CSF of chick embryos at the early stages is involved in regulating neuro-ectodermal cell behavior, such as cell proliferation and neurogenesis (6). IGF_2_ signaling likely promotes proliferation of progenitor cells during cerebral cortical development. Researchers have reported that IGF_2_ expression in the rat CSF was temporally dynamic with a peak during periods of neurogenesis and decline in adulthood (7). HGF promotes the survival and migration of immature neurons. After birth, there is a rapid increase in HGF expression until day P_2_ that coincides with migration of neurons and glial in the cerebral cortex (4). The high concentration of proteins in the E-CSF compared to the adult CSF suggests that the protein in this fluid may partake in brain development. One of the functions attributed to embryonic and fetal CSF proteins is the generation of osmotic pressure inside the embryonic brain cavity, which is necessary for expansion of the cerebral primordium (8, 9). The CSF plays a number of key roles in neural development during embryonic and early days after birth. These roles include regulation of survival, proliferation, and differentiation of neuro-epithelial progenitor cells (10). It has been demonstrated that the CSF acts as a growth environment for cortical progenitor cells where the age of both the CSF and stem cells interact to ensure that different parts of the CNS develop at the correct times (11).

Stem cells are characterized by long-term selfrenewal capability and the ability to differentiate into multiple cell types in response to induction factors (12). The adult bone marrow (BM) contains two separate populations of stem cells. The hematopoietic stem cells (HSCs) give rise to all other blood cells via hematopoiesis (13, 14). Mesenchymal stem cells (MSCs) (13) are the non-hematopoietic part of the BM that consist of various heterogeneous populations (15). They are called BM derived from MSCs (BM-MSCs) or BM mesenchymal stromal cells (16).

MSCs are multi-potential cells that reportedly reside in virtually all postnatal organs and tissues. BM-MSCs are clonogenic, non-hematopoietic stem cells that exist in the BM and have the capability to differentiate into multiple mesoderm-type cell lineages such as osteoblasts, chondrocytes, and endothelial cells as well as non-mesoderm-type lineages (neuronal-like cells) (17-19). BM-MSCs are defined by their ability to adhere to plastic and express specific sets of surfacecell markers (20, 21). The identification and selection of stem cells within a given tissue or organ mainly relies on the presence of specific surface cell markers- CD29^+^, CD44^+^, CD73^+^, CD90^+^, CD105^+^, CD146^+^, PDGFR^+^, CD31^-^, CD34^-^, CD45^-^, and Stro-1^-^ (14, 22). This investigation examines the differentiation of BMMSCs into neural cells by using E-CSF obtained at various gestational ages. This study aims to assess the ability of CSF to stimulate proliferation and neural differentiation of BM-MSCs for cell therapy applications.

## Materials and Methods

In this experimental study, wistar rats were bred inhouse at the Research Facility of the Department of Biology, Kharazmi University after the Animal Use Committee at Kharazmi University provided Ethical approval. The animals were maintained in large rat cages at a constant temperature on a 12-hour light/dark cycle starting at 8 am and with free access to food and water. For timed mating, the individual male and female rats were paired in mating cages and checked regularly for the presence of a vaginal plug, which was taken as an indication of successful mating. This day was noted as embryonic day zero (E0). At specific gestational time points, the pregnant dams were euthanized by cervical dislocation, the uteri rapidly removed and placed on ice, and the fetuses were dissected out onto ice. A pregnant rat usually has 10-15 fetuses.

### Collection of cerebrospinal fluid samples

We collected the CSF from the cisterna magna of the rat fetuses at E19, E20, and P1 using glass micropipettes and capillary action without aspiration. Samples containing undesirable blood contamination, visualized as a pink colour in the fluid, caused by damaging a blood vessel within the cisternal cavity, were discarded. All samples were collected into sterile microtubes and centrifuged at 4000 rpm. The clear supernatant was transferred into sterile tubes and stored at -40˚C. We used 200-300 pooled fetuses per age group for CSF analyses.

### Total protein analysis

Samples were pooled according to specific prenatal and postnatal ages. Total protein concentration was measured using the Bradford protein assay with absorbance measured at 595 nm wavelength.

### Preparation and culture of bone marrow mesenchymal stem cells

Male adult NMRI mice (6-8 weeks) were sacrificed, and we removed the femurs and tibias from both hind legs. After cleaning, the BM was flushed out with Dulbeccos’ modified Eagles’ medium (DMEM, Gibco, UK) using a syringe 0.5 ml with a needle. Cells were disaggregated by gentle pipetting through a decreasing needle bore size. The suspension of cells obtained was centrifuged at 1500 rpm at 25˚C for 5 minutes before resuspension in 1 ml of medium. Cells were seeded at a density of 10^4^ cells/cm^2^ in a 25 cm^2^ plastic flask in DMEM, 15% fetal bovine serum (FBS, Gibco, UK), 100 U/ml penicillin, and 100 mg/ml streptomycin (Gibco, UK) then incubated at 37˚C and 5% CO_2_. After 48 hours, we removed any nonadherent cells by replacing the medium. We added fresh medium every 3 or 4 days. Passage-2 cells were used for the eperiments.

### Differentiation of bone marrow mesenchymal stem cells

We induced adipogenic differentiation by culturing 90% confluent cultures in adipogenic induction medium that contained DMEM-LG with FBS, isobutylmethylanthine, insulin, deamethasone, and indomethacin. The medium was changed every third day. After 2 weeks, the cells were stained with oil red O to confirm adipogenic patterning (Sigma-Aldrich, USA). Osteogenic differentiation was confirmed after BM-MSCs were incubated in induction medium that included DMEM-LG with FBS, deamethasone, ascorbate, and β-glycerophosphate for approximately 14 days. In order to assess extracellular calcium deposits, the cultures were stained with 2% alizarin red S (Sigma-Aldrich, USA).

### Surface antigen analysis of bone marrow

mesenchymal stem cells by flow cytometry Pasage-3 adherent cells were treated with 0.25% trypsin (Gibco, UK) and washed twice with PBS. Cells were incubated with the antibodies mouse anti-CD44, mouse anti-CD73, and mouse anti- CD45 (Abcam, UK) for 30 minutes at 4˚C and resuspended in 100 μl of PBS. (Gibco, UK) Unbound antibodies were removed by washing with PBS. After washing, the cells were incubated for 40 minutes at room temperature in the dark using FITC conjugated secondary antibody and re-suspended in PBS for FACS analysis. At least 1×10^6^ cells per sample were analyzed with a flow cytometer.

### Bone marrow mesenchymal stem cell culture and in vitro tests

All eperiments were carried out in triplicate. Passage-2 cells were seeded, (about 7×10^4^ cells) in 24-well plates with media changes every 2-3 days. The attached cells were eposed to CSF (E19, E20, and P1) at 0, 3, 7, and 10% v/v concentrations in DMEM, 100 U/ml penicillin, and 100 mg/ml streptomycin. Cell morphology was eamined for neurite outgrowths after one week. Cells in individual wells were photographed and further analyzed using ImageJ software (NIH) (3). A neurite was defined as when the cellular process was longer than the diameter of the cell body. The average length of the neurites was calculated from measurements of 10 cells in each of 6 wells for each age group of CSF tested. We used b-FGF as a positive promoter of BM-MSC proliferation and differentiation into neuronal phenotypes to compare to the effects of CSF.

### MTT assay

Cell viability and/or proliferation were quantitatively determined by the 3-(4, 5-dimethyl 2 thiazolyl)-2,5- diphenyl 2 tetrazolium bromide (MTT) method. MTT is a yellow tetrazolium dye that responds to metabolic activity. Reductases in living cells reduce MTT from a pale yellow color to dark blue formazan crystals. In 24-well plates, the cells were seeded at 7×10^4^ cells/ well in 500 μL DMEM without FBS. Following attachment, the cells were eposed to CSF (E19, E20, and P1) at concentrations of 0, 3, 7, and 10% (v/v). After 24 hours, we added 100 ml of MTT (Merck, Germany, 5 mg/mL in PBS) to each well and the cells were allowed to incubate for 4 hours at 37˚C. Finally, after removal of the supernatant, 2 ml dimethylsulfoide (DMSO, Merck, Germany) was added to each well to dissolve the blue substance. Absorbance was read at 570 nm in disposable cuvettes. All eperiments were carried out in triplicate.

### Immunocytochemistry

The cells were fied in 4% paraformaldehyde in PBS for 15 minutes, permeabilized with 0.1% Triton -100 (Merck, Germany) for 30 minutes at room temperature, blocked with 5% BSA (Sigma-Aldrich, USA) in Tween 20 (Merck, Germany) in PBS (T-PBS, Gibco, UK) for 1 hour at room temperature, and then incubated at 4˚C overnight in the presence of either anti-ß-III tubulin (ABcam, UK, 1:100) or anti-MAP_2_ (ABcam, UK, 1:100). Negative controls, to verify the specificity of the antibodies, were obtained by omission of the primary antibodies, and incubating only with secondary antibodies. After three washes with T-PBS in PBS, we added FITC conjugated goat anti-mouse IgG (1:50, Abcam, UK) at room temperature for 1 hour. Cells were washed and photomicrographs were taken with a florescence microscope (Olympus, Japan).

### Statistical analysis

Statistical analysis was performed by one-way ANOVA and the Tukey test. P<0.05 were considered significant.

## Results

**Fig.1:**
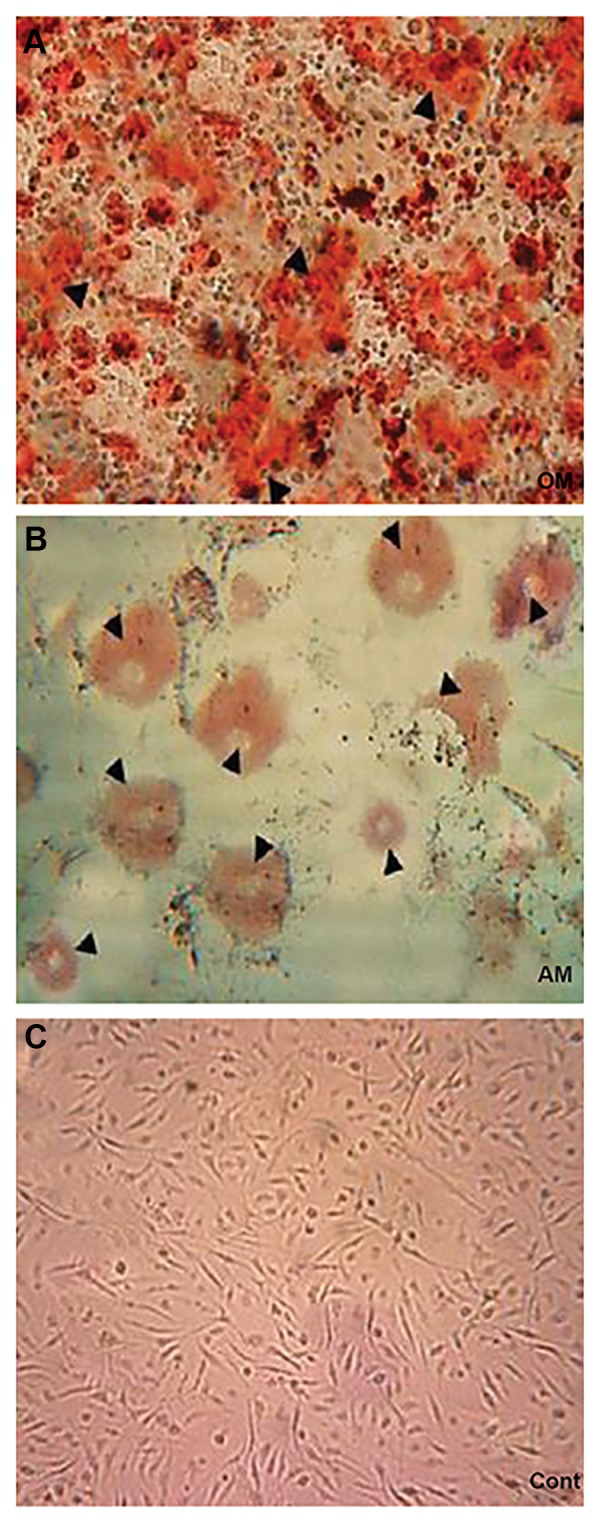
Characterization of bone marrow mesenchymal stem cells (BM-MSCs) and tests for multipotential characteristics. A. Cells grown in osteogenic medium (OM) medium with no defined cytokines to promote differentiation (×100), B. Cells grown in adipogenic medium (AM) stained with oil red O (×200). The open arrow heads show fat cells, and C. Cells grown in control medium (Cont) and stained with alizarin red (×100). The open arrow heads demonstrate mineralization of the extracellular calcium deposits.

BM-MSCs are typically isolated from BM based on their preferential adherence to plastic. After two or three passages, BM-MSCs became homogeneous in appearance with two distinct populations of large flattened cells and spindle-shaped cells (Fig .1A)

### Adipogenic and osteogenic differentiation

We induced adipogenic and osteogenic differentiation to confirm the differentiation potential of the BMMSCs. BM-MSCs showed adipogenic differentiation (lipid vacuoles stained with oil red O) after treatment for 2 weeks in adipogenic medium (AM, Fig .1B). Mineralization of the etracellular calcium deposits were assessed by alizarin red S staining (Fig .1C).

### Bone marrow mesenchymal stem cell surface marker expressions

**Fig.2:**
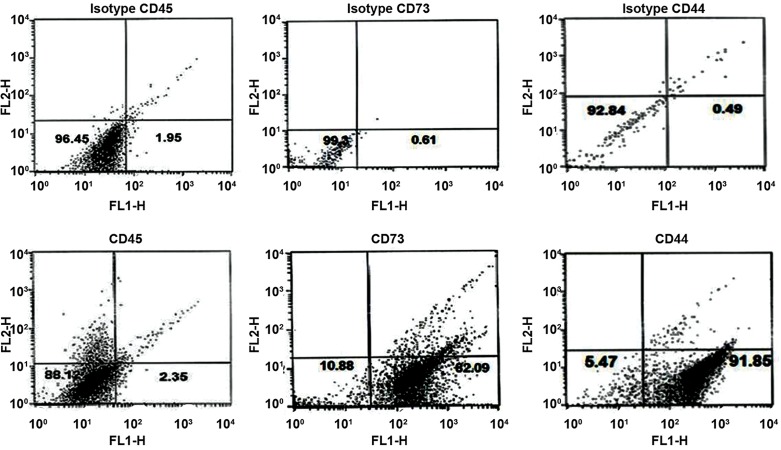
Flow cytometry analysis of bone marrow mesenchymal stem cell (BMMSC) surface markers. The BM-MSCs suspension was immunostained for CD44, CD73, and CD45. Cell surface analysis of BM-MSCs by FACS revealed that BM-MSCs were positive for CD44 and CD73 but negative for CD45.

Flow cytometry analysis of the P3 surface antigens revealed that the BM-MSCs were positive for CD44 and 73, and negative for CD45 (Fig .2).

### Total protein concentration

The CSF from E19 rat fetuses had a slightly higher mean total protein concentration (1.6 ± 0.1 mg/ml) compared to the E20 (1.5 mg/ml) and postnatal P1 (1.07 mg/ml) CSF.

### Neural differentiation of bone marrow mesenchymal stem cells induced by pre- and postnatal cerebrospinal fluid

**Fig.3:**
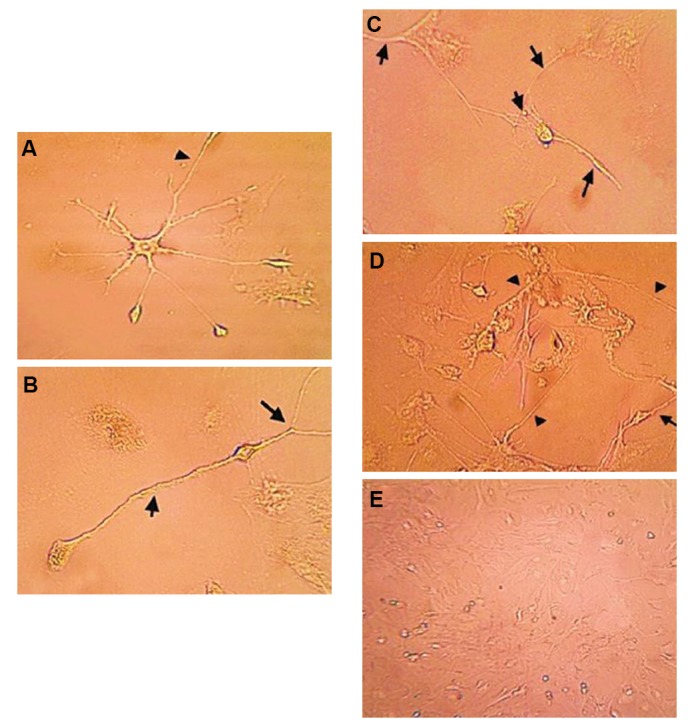
Bone marrow mesenchymal stem cells (BM-MSCs) after 7 days in culture with the addition of 10% pre- and postnatal cerebrospinal fluid (CSF) photographed with phase-contrast optics. A. Culture with E19 CSF (×400), B. Culture with E20 CSF (×400), C. Culture with P1 CSF (×400), D. Basic fibroblast growth factor (b-FGF, positive control) (×400), and E. Control group (×200) without pre- and postnatal CSF. There was significantly greater neural differentiation of BM-MSCS cultured in CSFsupplemented medium from E19 compared to E20 and P1.

Three days following CSF treatment, the spindle shaped BM-MSCs elongated and the neurites of the neuron-like cells became longer after 7 days. Inverse microscopic eamination of BM-MSCs showed the presence of neuron-like cells in the cell cultures treated with E19, E20, and P1 CSF and b-FGF (positive control) compared to the control group (without CSF, Fig.3.), BM-MSCS cultured in E19 CSF-supplemented medium had significantly greater neural differentiation compared to the E20 and P1 groups.

### Measurement of neurite outgrowth

**Fig.4:**
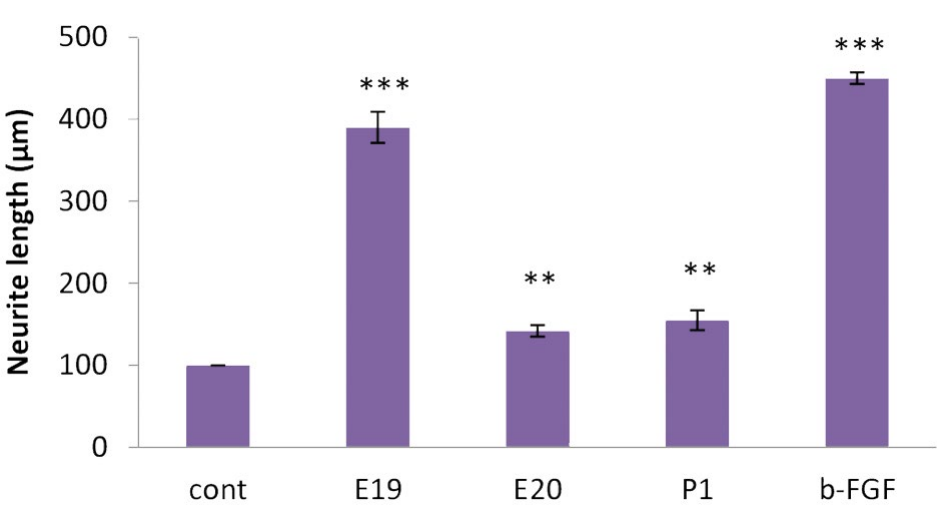
In vitro neurite growth from bone marrow mesenchymal stem cells (BM-MSCs). Cells were cultured for 7 days in media alone (cont) or media supplemented with 10% CSF from E19, E20, and P1 CSF or 10 ng/ml basic fibroblast growth factor (b-FGF) as the positive control. Neurite length was measured for at least 10 representative cells from each of 6 wells for each age of CSF tested. The E19 treated cells had greater density and neurite length compared to the E20 and P1 treated cells. **; P<0.01 and ***; P<0.001 compared to the control culture.

The cells had a significantly greater average neurite outgrowth compared to the controls when cultured in the presence of b-FGF (P<0.001), E19 (P<0.001), E20 (P<0.01), and P1 (P<0.01) for 7 days (Fig .4). Figure 4 shows that the length of the neurites in treated cells was much greater in the presence of b-FGF compared to CSF (E19, E20, and P1). b-FGF (positive control) promoted neurite outgrowth.

### Cell viability/proliferation

**Fig.5:**
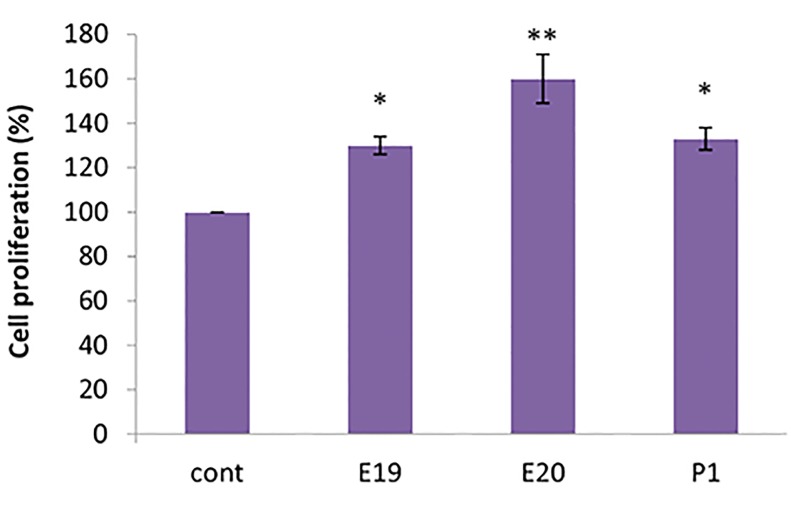
Cell proliferation of bone marrow mesenchymal stem cells (BM-MSCs) cultured in pre- and postnatal cerebrospinal fluid (CSF)- supplemented medium from the 10% concentrations of E19, E20, and P1. Results are expressed as a percentage of control levels. All cultures with added CSF had higher viability, which was significant with E20 CSF. *; P<0.05 and **; P<0.01.

Viability and cell proliferation of BM-MSCs cultured in CSF-supplemented medium from E20 rat fetuses had greater viability and cell proliferation compared to the other treated groups. We observed increased cell proliferation of BM-MSCs cultured in E19, E20, and P1 CSF at the 10% concentration compared to the 3 and 7% concentrations. We concluded that the effective CSF concentration for proliferation of BMMSCs was 10% (Fig .5).

### Immunocytochemical characteristics of differentiated bone marrow mesenchymal stem cells

**Fig.6:**
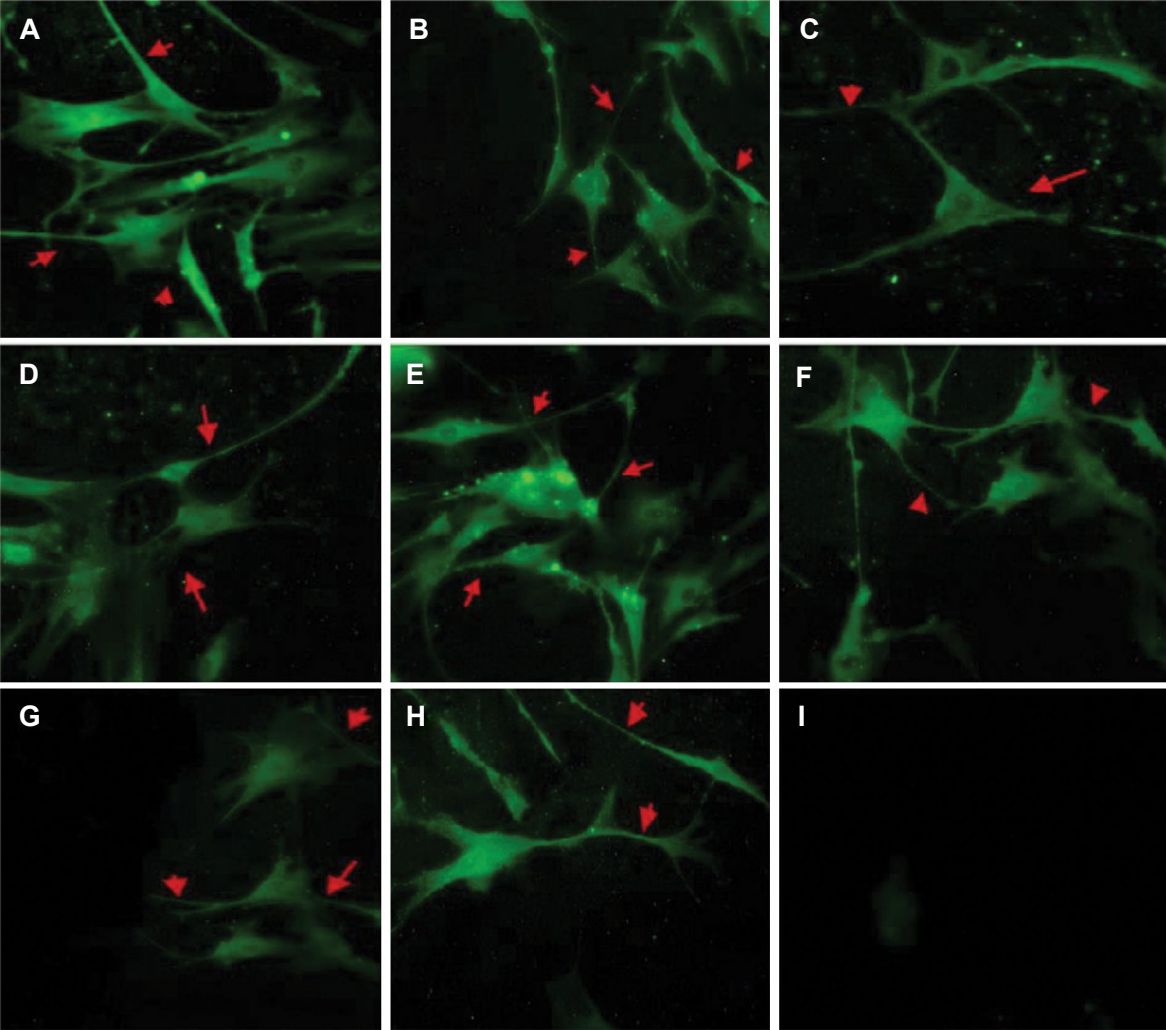
Cerebrospinal fluid (CSF) induced neuronal differentiation in the bone marrow mesenchymal stem cells (BM-MSCs). A. β-III tubulin expression in BMMSCs cultured with 10 ng/ml basic fibroblast growth factor (b-FGF) (×400), B. 10% E19 CSF (×400), C. 10% E20 CSF (×400), D. 10% P1 CSF (×400), E. MAP-2 expression in BM-MSCs cultured with 10 ng/ml b-FGF (×400), F. 10% E19 CSF (×400), G. 10% E20 CSF (×400), H. 10% P1 CSF (×400), and I. Control (×200) cultures without added CSF cells that show no stain when primary antibody was omitted. Red arrows indicate positive expression of β-III tubulin and MAP-2 in neuronlike cells and neurites. The highest expression level for these markers was detected in E19.

β-III tubulin and MAP-2 expressed in BM-MSCs in media supplemented with E19, E20, and P1 CSF, and in cells cultured with b-FGF (Fig .6).

## Discussion

It is widely agreed that stem cells are a substantial component of cell-based therapies as relatively high numbers of healthy cells are needed (15, 23). Currently, osteogenic and adipogenic differentiation are the most common methods used to identify and analyze cell populations and multi-lineage differentiation (24, 25). Our study results have demonstrated that BM-MSCs are capable of multi-lineage differentiation. The general strategy for identifying *in vitro* cultivated BM-MSCs is to analyze the epression of surface-cell markers such as CD44, CD45, and CD73. The FACS eperiments have indicated that BM-MSCs were positive for CD44 and CD73, and negative for CD45, a cell-surface marker associated with lymphohematopoietic cells (22). Therefore, we have observed no evidence of hematopoietic precursors in the cultures. Neurogenesis in the normal rat brain is a process that includes proliferation, migration, and differentiation. Days E19 and E20 coincide with migration of immature neurons and differentiation of migrated neurons (26). Studies show that undifferentiated cells migrate and neural differentiation form during the early postnatal stage (27). We have selected E19, E20, and P1 for CSF sampling.

In the present study, the E19, E20, and P1 CSF treatments induced BM-MSCs to differentiate into cells that had a neuronal phenotype and enhanced proliferation of BMMSCs relative to the control group. The most critical substances of the CSF are its protein components; their quality and quantity can change during CNS development (28).The present study has shown that CSF from E19 rat fetuses has a protein concentration of approximately 1.6 mg/ml which decreased to 1 mg/ml in P1 CSF. E19 has a high protein concentration compared to other age groups, whereas P1 has the lowest protein concentration. Total protein in CSF increased from birth to a peak concentration between 5 and 10 days, after which it declined rapidly (29). Growth factors are important for development of the cerebral corte, including FGF, TGF-β, NGF, BDNF, NT- 3, IGFs which are found in fetal CSF. Proteomic studies have shown the presence of mitogenic factors in CSF (30). Based on evidences, the CSF plays an important role as a neural stem cell niche and provides a microenvironment for regulation of neuroepithelial cells (31). The proteomic composition of fetal CSF suggests that it contains all of the secretory factors, growth factors, cytokines, etracellular matriproteins, and adhesion molecules, as well as numerous other materials and nutrients. These components are sufficient to maintain neural stem cell survival, and promote proliferation and differentiation of the progenitor cells into mature cells (32). Studies have reported great similarities in the composition of proteins in mammalian CSF such as humans, rats, and mice (6).

We hypnotized that the addition of different concentrations of CSF (E19, E20, P1) into the culture media would enable a better microenvironment to induce neural differentiation of BM-MSCs. The experimental groups had greater absorbance values compared to the control group, which indicated the improvements in cell proliferation and viability of BM-MSCs. These results demonstrated that prenatal and postnatal CSF had the potential to induce differentiation under *in vitro* culture conditions. In this study, we observed that β-III tubulin and MAp_2_ expression significantly increased in BM-MSCs cultured with CSF-supplemented medium compared with the control group. Based on these evidences, CSF promoted neuronal differentiation and proliferation of BM-MSCs in an age-dependent manner.

The *in vitro* survival, proliferation, and neuronal differentiation of BM-MSCs depend on certain growth factors which must be present in the CSF in order to obtain the effects observed in this study (11). Our knowledge about the role of the CSF in brain development and details of CSF functions helped us to understand how normal brain develop and to develop strategies and treatments to prevent neurodevelopmental abnormalities and neuropathological conditions. We concluded that regulation of CSF-born growth factors might be involved in some neurodegenerative diseases, such as MS and Alzheimers’ (33). We have provided evidence which suggests that the CSF induces proliferation and neural differentiation of BM-MSCs in an age-dependent manner. However, further eperiments are needed to identify effective CSF components in neural differentiation of BM-MSCs.

## Conclusion

Our results demonstrated that stimulatory effects of CSF on BM-MSCs can altered in an age-dependent manner. This finding confirms that the CSF is a powerful growth medium which promotes brain development. The CSF provides a rich environment for differentiation of BMMSCs *in vitro*. The functions of the CSF components and their role in BM-MSC differentiation should be further researched to improve cell therapy.
